# Novel nectar robbing negatively affects reproduction in *Digitalis purpurea*

**DOI:** 10.1002/ece3.8068

**Published:** 2021-08-31

**Authors:** Christopher R. Mackin, Dave Goulson, Maria Clara Castellanos

**Affiliations:** ^1^ School of Life Sciences University of Sussex Brighton UK

**Keywords:** bumblebee, *Digitalis purpurea*, nectar robbing, pollination

## Abstract

With many plant–pollinator interactions undergoing change as species’ distributions shift, we require a better understanding of how the addition of new interacting partners can affect plant reproduction. One such group of floral visitors, nectar robbers, can deplete plants of nectar rewards without contributing to pollination. The addition of nectar robbing to the floral visitor assemblage could therefore have costs to the plant´s reproductive output. We focus on a recent plant colonist, *Digitalis purpurea*, a plant that in its native range is rarely robbed, but experiences intense nectar robbing in areas it has been introduced to. Here, we test the costs to reproduction following experimental nectar robbing. To identify any changes in the behavior of the principal pollinators in response to nectar robbing, we measured visitation rates, visit duration, proportion of flowers visited, and rate of rejection of inflorescences. To find the effects of robbing on fitness, we used proxies for female and male components of reproductive output, by measuring the seeds produced per fruit and the pollen export, respectively. Nectar robbing significantly reduced the rate of visitation and lengths of visits by bumblebees. Additionally, bumblebees visited a lower proportion of flowers on an inflorescence that had robbed flowers. We found that flowers in the robbed treatment produced significantly fewer seeds per fruit on average but did not export fewer pollen grains. Our finding that robbing leads to reduced seed production could be due to fewer and shorter visits to flowers leading to less effective pollination. We discuss the potential consequences of new pollinator environments, such as exposure to nectar robbing, for plant reproduction.

## Graphical abstract

We test whether the addition of nectar robbers into the environment of a flowering plant (*Digitalis purpurea*) can lead to costs for reproduction. Following experimental robbing, we found a significant decline in the visitation rate and visit duration of the bumblebee pollinators, as well as a reduction in the number of seeds produced by fruits. The addition of new floral visitors to plant systems is likely to happen more often as humans influence the ranges of plants and pollinators, and here, we show how these changes can come with costs to the reproduction of the plant.

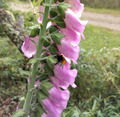

## 1 INTRODUCTION

Many plant–pollinator interactions are undergoing change due to multiple anthropogenic influences (González‐Varo et al., 2013; Goulson et al., 2015). At present, we have a limited understanding of how novel interactions affect plant reproductive success, for example, after plant invasion (Barrett et al., 2008; Chalcoff et al., 2019; Richardson et al., 2000). One interaction with consequences for plants is nectar robbing, where plants have their mutualism with pollinators bypassed by floral visitors (robbers) that consume nectar rewards without pollinating (Irwin et al., 2010). In the same way as pollination mutualisms, this interaction between plants and nectar robbers can also be altered as a consequence of changes in abundance or distributions of the plants or floral visitors (Irwin & Maloof, 2002; Traveset et al., 1998). In turn, changes in the incidence of nectar robbing have the potential to affect a plant´s reproductive success.

During nectar robbing, a floral visitor bites a hole in the corolla (“primary robbing”) or utilizes an existing hole previously created by another robber (“secondary robbing”) to feed from nectar, which often results in no contact with the stigmas or anthers and hence no contribution to pollination (Inouye, 1983; Rojas‐Nossa et al., 2016). Some previous studies found limited or no negative fitness consequences of robbing for the plant (Richman et al., 2018; Stout et al., 2000) with some examples of robbing increasing plant fitness through increasing pollen flow and dispersal distance (Higashi et al., 1988; Maloof & Inouye, 2000) and increasing the frequency of visitation from legitimate pollinators (Stout et al., 2000). However, other studies have reported detrimental effects on at least one component of the plant's reproductive success (Adler et al., 2016; Burkle et al., 2007; Castro et al., 2008; Irwin & Brody, 1999; Lara & Ornelas, 2001). Negative effects of robbers include damage to the reproductive organs, a reduction of the attractiveness of the floral display, and exhaustion of the nectar reward, all of which could potentially alter the foraging behavior of legitimate pollinators that are required for plant reproductive success (Irwin et al., 2010). The extent to which robbers affect plant fitness could depend on the frequency, the amount of damage done, and how much the behaviors of the legitimate pollinators are affected (Adler et al., 2016; Irwin et al., 2010). Additionally, if extra resources are allocated toward nectar production in the plant in response to robbing, this could have a detrimental effect on the number of seeds and/or fruits produced (Navarro, 2001; Pyke, 1991; Southwick, 1984). In this study, we focus on the effects of robbing on a plant that, after range expansion, experiences a high level of nectar robbing that is not present in the native range.

**FIGURE 1:**
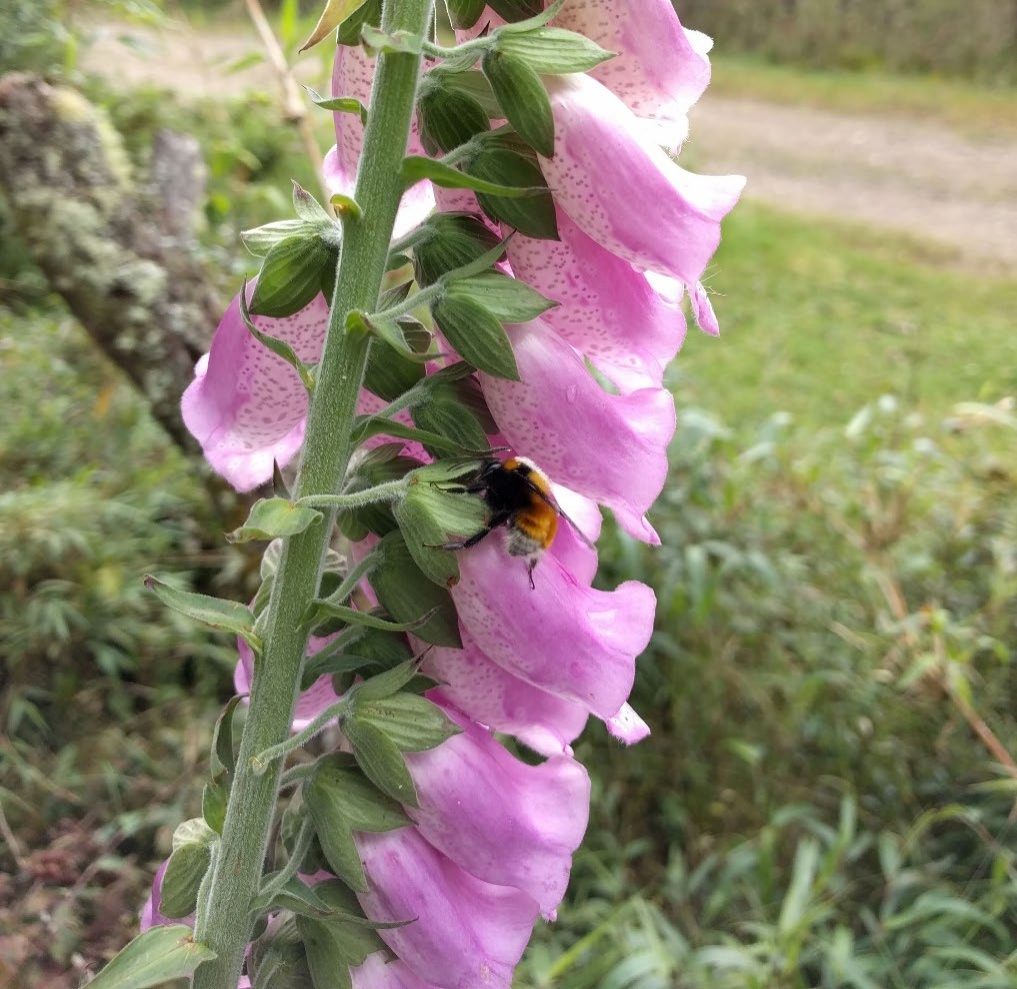
*Bombus hortulanus* worker robbing nectar from a hole at the base of *Digitalis purpurea* flowers in a non‐native population in Colombia. Photograph by MC Castellanos

The common foxglove, bumblebee‐pollinated *Digitalis purpurea* L. (Plantaginaceae), expanded its range from native European woodland to areas including tropical mountains in Central and South America following anthropogenic introductions (Mackin et al., 2021). As a consequence, the plant now experiences geographically variable rates of nectar robbing. The pollination biology of this species is well known in the native range, yet to our knowledge, only one record exists of nectar robbing in populations across Europe, associated with robbing specialist *Bombus wurflenii* (Reinig & Rasmont, 1988), whereas in American populations we found that the plants are robbed at a high rate (Figure 1). For example, in preliminary observations in 110 plants across two non‐native populations in Colombia, we recorded that 288 out of 677 (42.5%) recently opened flowers had been robbed at least once (pers. obs.). In these populations, the bumblebees *Bombus hortulanus* and *B. rubicundus,* and additionally, some species of hummingbird and flower piercers (specialized robbers in the genus *Diglossa*) frequently feed on nectar from *D. purpurea* by robbing the flowers. In Costa Rica, the high‐altitude bumblebee *B. ephippiatus* is the main pollinator of the plant but also a frequent robber. Frequently, individual bumblebees use a mixed foraging strategy on *D. purpurea*, robbing and visiting legitimately on the same foraging bout.

Here, we test the costs of novel levels of nectar robbing on *D*. *purpurea* by experimentally robbing plants and measuring the effect on pollinator behavior and on the plant's reproductive success. We expect nectar robbing will reduce the volume of nectar or alter the rate of nectar production, causing bees to visit flowers at a lower rate (Parachnowitsch et al., 2019). Additionally, it is possible that, through increased replenishment of nectar, robbing could also reduce energetic resources available for fruit production, regardless of effects on bee visitation (Navarro, 2001). We measured both male and female components of reproduction for plants and quantified the visitation rates by naturally foraging bumblebees to each treatment. We also recorded other measures of bumblebee visitation patterns on inflorescences, including time spent visiting flowers and proportion of an inflorescence's flowers that were visited in a foraging bout.

## 2 MATERIALS AND METHODS

### 2.1 Study system and experimental setup

The facultative biennial herb *D. purpurea* (Plantaginaceae) produces long showy inflorescences (Figure 1) of nectar‐rich flowers that open in sequence from the bottom up. The flowers are protandrous, with anthers dehiscing following anthesis and the stigma becoming receptive three to five days after anthesis. Animal pollination is required to achieve full seed set (Mackin et al., 2021; Nazir et al., 2008), and the primary pollinators in the native range are the long‐tongued bumblebees *Bombus hortorum* and *B. pascuorum* (Broadbent & Bourke, 2012; Grindeland et al., 2005). In introduced populations in Central and South America, hummingbirds are also important pollinators, but several species of *Bombus* remain the most frequent ones (Mackin et al., 2021). In those populations, we also observe high levels of nectar robbing by bumblebees (Riveros et al., 2006), *Diglossa* flower piercers, and hummingbirds. We have not observed bumblebees making holes on the corollas; instead, bees and hummingbirds are likely secondary robbers using the holes pierced by *Diglossa* birds. In surveys in two localities in Colombia (Floresta *N* = 50 and Choachí *N* = 60), we found that 36.2% and 47.8% (respectively) of all recently opened flowers were robbed, with robbing making up to 14.1% to 19.4% of all visits to flowers in those two populations.

Nectar production and secretion begins the day before the first pair of anthers dehisce, and peaks during anthesis and stigma maturation (Percival & Morgan, 1965). Nectar is produced in floral nectaries located at the base of the ovary and escapes through modified stomata that are permanently open (Gaffal et al., 1998). Nectar sugar concentration ranges from 16%–27% and is predominately made up of sucrose (78.5%) with some glucose and fructose sugars (Gaffal et al., 1998). In our study populations, a single flower secretes between 3.1 and 10.5 µl of nectar over 24 hr without visitation (pers. obs).

We grew *D*. *purpurea* potted plants from seed collected from a wild population near Portsmouth (UK) in 2017 that were grown until flowering in summer 2019 and then transported to the University of Sussex campus in Falmer. Between 16 and 18 plants were selected at random to be in one of two treatment groups—“robbed” and “control” (non‐robbed flowers). To standardize the rate of natural pollination that all plants experienced, plants were exposed for 3 hr each day to receive visits by local bumblebees, and for the other period of 21 hr, the inflorescences were covered with a mesh bag to block visitation.

Plants in the robbed treatment group had all recently opened flowers manually robbed by piercing a hole in the proximal corolla tube with a microcapillary tube twice during the 3‐hr pollination period (once at the start and then again after 90 min). This rate of robbing is based on field observations in the non‐native range where *D. purpurea* flowers are robbed less than once per hour, and on greenhouse trials on the experimental plants that showed that nectar is replenished at a rate of 2.3 µl per hour during three hours following nectar depletion (*N* = 30 flowers over 10 plants). Compared to other species tested, this is a fast rate of replenishment (Castellanos et al., 2002) as a single foxglove flower secretes up to 10.5 µl of nectar per day.

Bumblebees will often avoid plants that have recently been visited by other floral visitors using olfactory and other cues (Stout et al., 2000), so we manually handled the inflorescences (both controls and robbed) to mimic contact during simulated robbing. This entire procedure was repeated daily for the period that plants flowered between 17 June and 3 July 2019.

### 2.2 Bee behavior and visitation rates

Apart from the robbing behavior, *Bombus* species in the native and non‐native ranges behave very similarly when visiting foxgloves flowers. In this experiment, we focus on native bumblebees to understand how robbing affect bumblebee pollination in general. To test whether robbing leads to altered behavior of bumblebees, we recorded several aspects of visitation. First, we tested whether robbing would cause a change in visitation rates, by quantifying the number of visits per flower per hour on plants in both treatments when in full bloom. We also recorded the identity of bumblebee species that visited when conducting censuses. Plants were arranged in a line, separated by about 1 meter, with alternating treatments and the individuals within each treatment had their positions randomized. We recorded counts of each bumblebee species visiting flowers on control and robbed treatment plants and used 644 3‐min censuses to quantify visitation rates in 3‐hr periods that covered different times of the day when floral visitors were active. Second, we also measured potential effects of robbing on the duration of floral visits by bumblebees. We recorded with a stopwatch the length of visits to individual flowers, defined as the time between entering a flower to feed and appearing at the mouth of the corolla to exit it.

Bumblebees typically visit several flowers on a foxglove inflorescence in each foraging bout, so to test whether robbing had an effect on a continuous foraging bout we measured the proportion of flowers on an inflorescence that individual bumblebees visited per foraging bout. Finally, we also recorded the number of times a plant in a treatment was “rejected,” defined as a bumblebee hovering near flowers and leaving without landing, or landing on the flower and leaving without entering.

### 2.3 Effects on reproductive success

To test whether plant reproductive success was impacted as a result of nectar robbing, we estimated male and female components of reproductive output. For this, we measured the pollen export and number of seeds produced on average by experimental plants and then compared between treatments to test for differences between robbed plants and control plants.

#### 2.3.1 Pollen export

We used pollen export as a proxy for the male component of reproductive success, by quantifying the number of pollen grains removed from an anther by visitors in a 3‐hr period. In *D*. *purpurea* flowers, two anterior anthers are paired with each other and tend to dehisce simultaneously (Percival & Morgan, 1965), as do the two posterior anthers further inside the flower. To find the number of pollen grains removed (= pollen export), we subtracted the number of pollen grains left on a “postvisitation” anther from the number on a “previsitation anther” on the same flower. For this, immediately before the beginning of each allotted visitation period (whereupon inflorescences are still bagged), we collected one of the near‐dehisced posterior anthers (the “previsitation” control anther) in a centrifuge tube. Once dehisced in the tube, we added 70% ethanol until counting. We collected the corresponding anterior “postvisitation” anther (which was already dehisced before the visitation period begun) from the same flower immediately after the visitation period of 3 hr and stored it in 70% ethanol. We did not record the number of visits to “postvisitation” anthers, but each flower is likely to have received multiple visits. This was repeated for each plant on separate days to include 2–6 anther comparisons per plant for at least 12 plants per treatment.

We used a hemocytometer counting chamber to estimate the number of pollen grains on the “previsitation” and “postvisitation” anthers. Up to 30 min prior to counting, tubes containing anthers were sonicated for four lots of 30 s to dislodge pollen grains and to homogenize the grains in the ethanol. Immediately before counting, the ethanol–pollen mixture was then vortexed for five seconds and 8µl of the sample was pipetted into the chamber for counting. Pollen grains were counted in grid squares of a known volume (0.1 µl) and multiplied up for an estimate of the total number of pollen grains in the original 200 µl sample. We repeated this process four times for each sample and averaged for analysis. We calculated total pollen export by subtracting the number of pollen grains estimated to be left on a postvisitation anther from the number of pollen grains estimated to be on the corresponding paired previsitation anther.

This method assumes that the number of grains in anthers within a flower is similar. To test this, we counted the number of grains in all four anthers of eight flowers from different individual plants using the same method as above. We found that the anterior anthers did not produce significantly different numbers of pollen grains to the posterior anthers in a flower (*p* =.451; *N* = 32 anthers). This confirms that anthers in a given flower can serve as good previsitation controls in our experiment.

#### 2.3.2 Seed production

We used seed production per fruit as a proxy for the female component of plant reproductive success. Flowers on inflorescences received a daily three‐hour “window” of pollination by natural bumblebee visitation for the entirety of their flowering period. After the flowering had finished, we left plants unbagged to allow fruits to develop normally. Around 4–6 weeks after flowering, we collected between three and seven near ripe but undehisced fruits from each plant at various points on the inflorescence and then left them to dry inside paper envelopes to allow natural dehiscence of fruits. We photographed seeds on filter paper and then counted using a macro in ImageJ software.

### 2.4 Statistical analyses

Visitation rates (visits per flower per hour) were compared between robbed and non‐robbed plants using generalized linear models with a binomial distribution (flowers visited vs. not visited) in the *stats* package in R (R Core Team, 2020). We included the pollinator species as a fixed factor in the model to test for differences between the two bumblebee species, and a term for the interaction between treatment and pollinator.

The duration of a visit to a flower was compared between robbed and non‐robbed plants using linear models in R, where visit duration was log‐transformed as the data showed a skewed distribution. We included pollinator as a fixed variable in the model, to test for differences in visitation by the two species, and for whether there was an interactive effect of treatment and pollinator.

We tested for differences between control and robbed treatment plants for the proportions of flowers visited (with pollinator species included as a fixed factor) and the proportion of rejections using generalized linear models with a binomial distribution in the *stats* package in R.

We compared pollen export between the robbed and control treatments using mixed effects linear models in R, with plant individual as a random factor (packages *lme4* and *lmerTest,* Bates et al., 2015). We used the same approach to compare the number of seeds produced per fruit between the treatments.

## 3 RESULTS

### 3.1 Bee behavior and visitation rates

**FIGURE 2:**
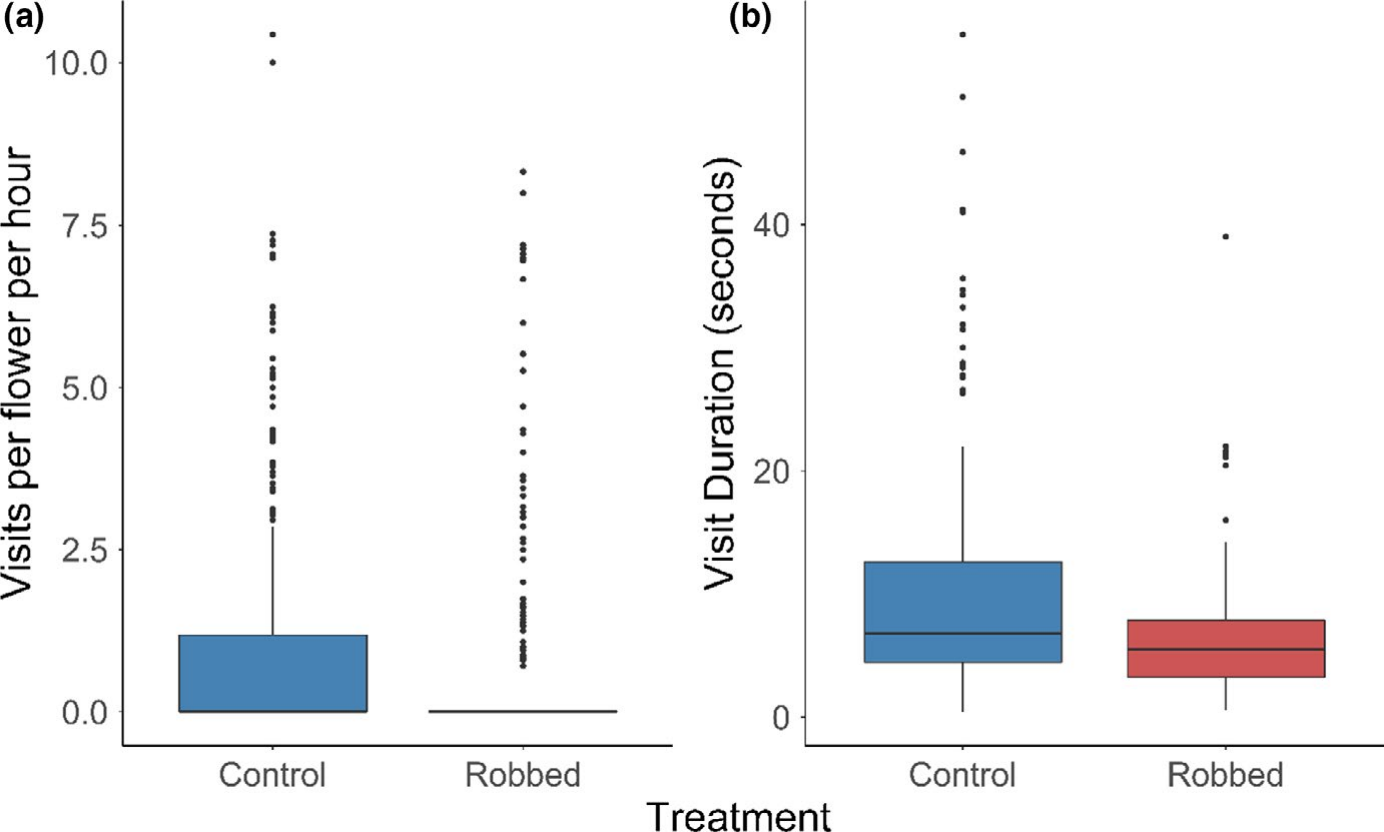
(a) The mean number of visits per hour to flowers by all bumblebees to non‐robbed control (blue, *N* = 322) was significantly higher than to robbed plants (red, *N* = 318; *p* < .001). (b) The mean flower visit duration by all bumblebees to non‐robbed control (blue, *N* = 210) was significantly higher than to robbed plants (red, *N* = 120; *p* < .001)

As expected, two bumblebee species were the only pollinator visitors to our experimental plants, with *Bombus hortorum* visiting flowers significantly more often (mean = 0.6 ± 1.6 *SD* visits per flower per hour) than *B. pascuorum* (mean = 0.2 ± 0.9 *SD*; *p* < .001; *N* = 1,288 3‐min surveys). Overall, robbed flowers received visits at a significantly lower rate (mean = 0.7 ± 1.7 *SD* visits per flower per hour) than flowers in the control treatment (mean = 1.0 ± 2.0; *p* < .001; Figure 2a), and this was consisted for both bumblebee species. *B. hortorum* visited control flowers and robbed flowers at rates of 0.6 ± 0.7 *SD* and 0.7 ± 1.7 *SD* visits per flower per hour (respectively) while *B. pascuorum* visits control and robbed flowers at rates of 0.2 ± 0.7 *SD* and 0.3 ± 1.0 *SD* visits per flower per hour (respectively).

**FIGURE 3:**
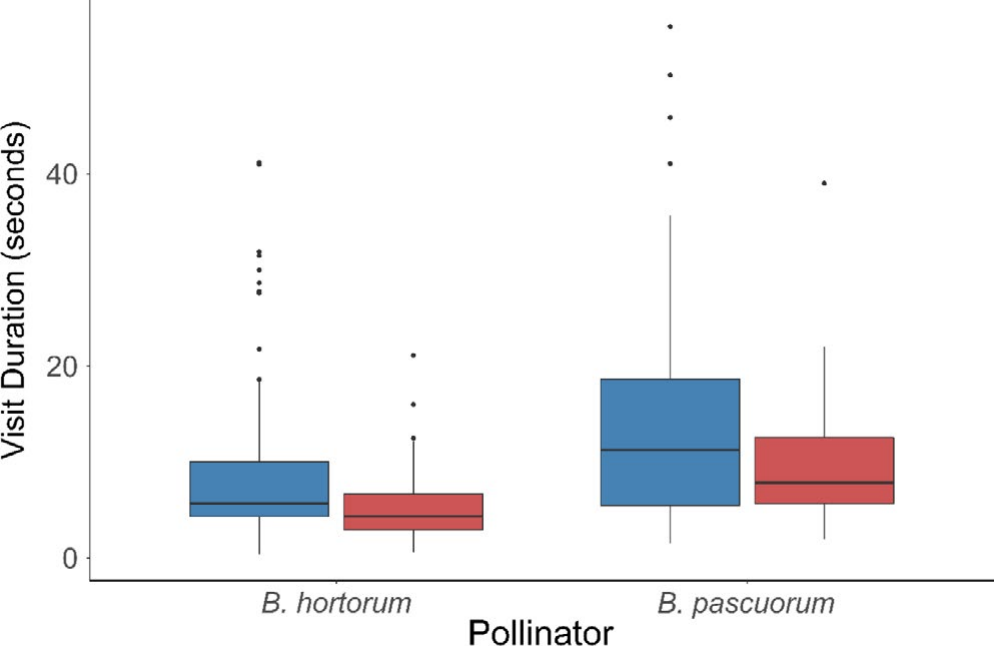
The mean visit duration to flowers by *Bombus hortorum* (*N* = 147) was significantly shorter than visits by *B*. *pascuorum* (*N* = 63; *p* < .001) to non‐robbed control (blue) and robbed (red) plants

We found that the average visit length to flowers was also different when comparing robbing treatments. Visits to robbed flowers were significantly shorter (mean = 6.6 ± 5.3 *SD* seconds; *N* = 120) than on the control flowers (mean =10.3 ± 9.5 *SD*; *N* = 210; *p* <.001), with visits being on average 3.7 s shorter (Figure 2b). This reduction in visit length was consistent across bumblebee species (Figure 3); however, visits by *B. hortorum* were overall of shorter duration (mean = 7.3 ± 6.3 *SD* seconds; *N* = 232) than *B. pascuorum* (mean = 12.9 ± 11.2 *SD*; *N* = 98; *p* < .001; Figure 3).

Bumblebees visited on average 50% of a control plant's flowers on an inflorescence (*N* = 52) and 42% of a robbed plant's flowers (*N* = 40), and this difference was statistically significant (*p* = .037). When recording the number of times that plants in each treatment were rejected by foragers, robbed plants were rejected 32 times compared with 25 times for the control; this was not significantly different (*p* =.090).

### 3.2 Effects on reproductive success

**FIGURE 4:**
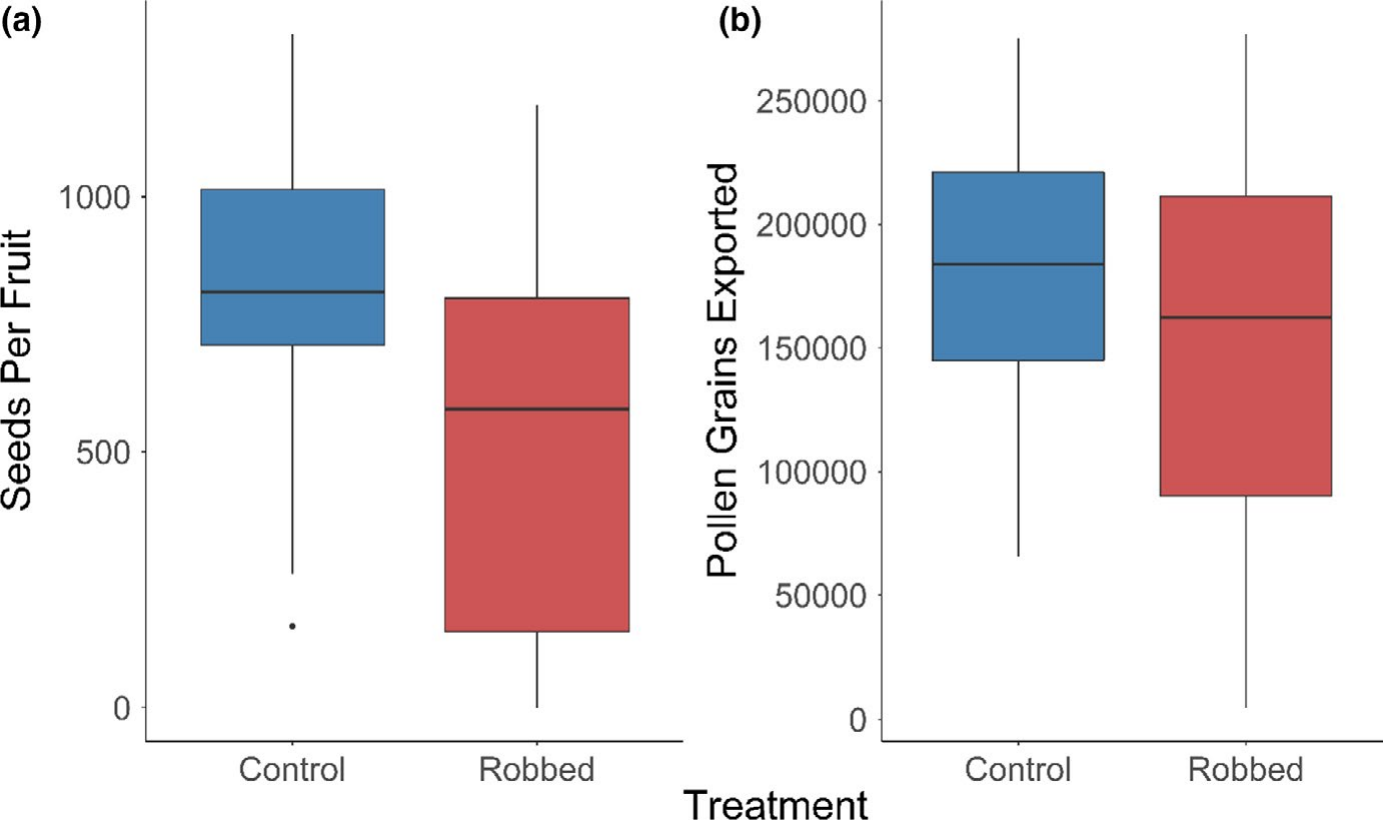
(a) The mean number of seeds produced per fruit in the non‐robbed control (blue, *N* = 85) was significantly smaller than in robbed plants (red, *N* = 59, *p* < .001). (b) There were no differences between the number of pollen grains exported from anthers in non‐robbed control (blue, *N* = 43) and robbed (red, *N* = 50; *p* = .141) plants

Fruits in control plants produced significantly more seeds (mean = 823.6 ± 48.98 *SD*; *N* = 87 fruits) than those in robbed plants (mean = 510.7 ± 71.18 *SD*; *N* = 72; *p* < .001; Figure 4a), with robbed plants producing 25% fewer seeds on average compared with control plants. In contrast, export of pollen grains was not significantly different between the robbed (149,167 ± 72,742 *SD* pollen grains, *N* = 50) and the control (178,329 ± 57,904 *SD*, *N* = 43) treatments (*p* = .141; Figure 4b).

## 4 DISCUSSION

With our experimental approach, we show how the addition of high levels of nectar robbing can have a cost to the reproductive output of a plant colonizing a new area. This cost is in terms of seed production, with robbed plants producing 25% fewer seeds compared with non‐robbed controls. Below, we discuss how this result can be related to changes in pollinator behavior and the potential implications of these costs for the naturalized populations exposed to novel levels of robbing.

Visiting bumblebees altered their behavior when interacting with robbed plants by having a significantly reduced visit rate and visiting a smaller proportion of flowers in the inflorescence compared to non‐robbed plants. This is consistent with other experimental studies removing nectar (Li et al., 2021) and with the fact that bumblebees tend to spend less time on an unrewarding inflorescence, where departure can be triggered by encountering one or more unrewarding flowers (Best & Bierzychudek, 1982; Heinrich, 1979). We also found that bumblebees reduced the length of floral visits on robbed flowers compared with non‐robbed flowers. This could potentially be due to a lower volume of nectar present in robbed flowers taking less time to drink and bumblebees leaving sooner (Hodges & Wolf, 1981). Richardson (2004) also found that bumblebee visit duration was reduced in robbed flowers compared with unrobbed flowers. Other studies have found that robbing is associated with reduced visitation rates (Irwin et al., 2010), and some suggest that robbing holes and damage to the flower are off‐putting to visitors (Goulson et al., 2007; Varma et al., 2020; although this is not always the case (Souza et al., 2019)). In our experiment (as in non‐native field populations of *D. purpurea*), holes were made at the base of the corolla, which in field conditions may be out of visual range for legitimate visitors to detect and be repelled by, especially for the fast paced foraging of hummingbird pollinators. However, since there is evidence that hummingbirds can use visual cues to discriminate against robbed flowers (Lara & Ornelas, 2001), it would be interesting to assess to what degree hummingbird pollinators in the introduced range are dissuaded from pollinating by the robbing holes as well as from a lack of nectar resulting from robbing (Irwin, 2000).

The two visitors to our experimental plants were *Bombus hortorum* and *B. pascourum*, with the former visiting three times more often, consistent with what is observed in nature in the UK (Broadbent & Bourke, 2012; Mackin et al., 2021). Interestingly, *B. pascuorum* visited individual flowers for a longer duration, regardless of robbing treatment. This could be explained in part by the shorter tongue length of *B. pascuorum*, causing them difficulty feeding in *D. purpurea* flowers and so they take a longer time to complete a visit. In any case, both bumblebee species showed the same patterns of reduced visitation to robbed flowers compared to non‐robbed ones.

The change in bumblebee behavior could be contributing to the lower reproductive output we find for the female component of reproduction in robbed plants, with intense levels of nectar robbing causing less frequent and shorter visits which ultimately reduces pollen deposition and therefore seed production. This idea is supported by several studies that find the duration of visits by bumblebees positively correlates with pollen deposition (Cresswell, 2000; Kudo, 2003; Thøstesen & Olesen, 1996). This is not always the case; in a study by Richardson (2004) bumblebees spent less time in robbed flowers but visit duration did not correlate with amount of pollen deposited. Other authors finding similar results to ours (Irwin & Brody, 1999; Lara & Ornelas, 2001), suggest that reduced attractiveness of flowers can lead to a reduction in the pollinator visitation rate and a lower seed production. However, we cannot rule out the possibility that resource depletion resulting from manual robbing also contributed to the reduced seed production. This could be caused by increased metabolic costs for the plants associated with increased production of nectar (Doust & Doust, 1988; Navarro, 2001). Future work could use pollen supplementation to distinguish between the effects of changes in nectar robber behavior from metabolic costs of nectar on reproductive output.

In contrast to findings we present here, many studies suggest robbing can have limited or no negative effects on the female component of fitness (Andalo et al., 2019; Carrió & Güemes, 2019; Maloof, 2001; Richardson, 2004; dos Santos et al., 2020; Varma et al., 2020; Varma & Sinu, 2019; Zimmerman & Cook, 1985). This lack of an effect on reproductive output could be due to the legitimate pollinators still visiting the plant and saturating the stigmas with enough pollen so the plant can achieve full seed set (Heiling et al., 2018; Stout et al., 2000). One potential caveat in this study is that our ability to detect differences in mean seed production could be biased by the standardized three‐hour visitation periods per day in our experiment. This is because open visitation during the lifetime of the flower could lead to full seed set, even with a reduced visitation rate. However, even with this restriction on the amount of visits plants could receive, these experimental results are consistent with the comparatively low seed set we have observed in the non‐native populations (as we discuss below).

We found that nectar robbing did not negatively affect the male component of reproduction through pollen removal by bumblebees. Other studies found that nectar robbing can include a cost to the male component of fitness in some species (Castro et al., 2008; Irwin & Brody, 1999; Irwin & Maloof, 2002; Richardson, 2004) but not in others (Maloof, 2001; Morris, 1996; Richman et al., 2018). With the method we used here, it is unclear how much of the pollen released from anthers ultimately reaches stigmas of conspecific plants. As with using any proxy as a measure of reproductive success, in this case it is difficult to deduct the entire picture as to whether nectar robbing affects male success.

The aim of our experiment was to simulate the conditions of nectar robbing on potted plants of *D. purpurea*, with the idea that similar effects could be found for plants in the non‐native robbed field populations. The reduced seed production following addition of nectar robbing we observe here is consistent with our previous observations showing that non‐native plants in populations with nectar robbers have a significantly lower lifetime seed production (average = 40,788 ± 20,644 *SD* seeds, across three populations in Colombia and Costa Rica; *N* = 211 plants) compared with native populations with no robbing (average = 113,812 ± 84,868 *SD* seeds across two populations in the UK; *p* < .001; see also Mackin et al., 2021). Although many other factors could be involved, the high levels of nectar robbing could be contributing to the lower average reproductive output in the introduced range. In pollinator surveys in the same naturalized populations in Colombia, we found that individual *Bombus hortulanus* and *B. rubicundus* bumblebees used a mixed strategy of visiting flowers both legitimately and robbing. This can be common in robbing interactions (Morris, 1996) although often bumblebees adhere to a consistent strategy to reduce handling time during a foraging bout (Bronstein et al., 2017). If plants are already receiving adequate pollination, then nectar robbing may only incur reproductive costs if a certain threshold of robbing intensity is reached that depletes nectar or alter visual cues enough to deter legitimate visitors (Irwin et al., 2015).

With the intensity of nectar robbing varying across populations so radically, there could be considerable differences among populations in robber‐mediated selection on floral traits (Castro et al., 2008; Navarro & Medel, 2009). Plant populations experiencing a high level of robbing could evolve local resistance or tolerance to nectar robbing (such as phenological, mechanical, or chemical barriers) even at the cost of decreasing the attraction to pollinators and reducing reproductive output compared with other populations (Adler et al., 2016). It is intriguing that native *D. purpurea* populations experience low levels of nectar robbing, even in the presence of bumblebee species that are capable of making holes and often rob other plant species (*Bombus terrestris, B*. *lucorum, and B. wurflenii*). *D. purpurea* plants produce high levels of toxic cardenolide compounds (Evans & Cowley, 1972) that are also present in the nectar (Palmer‐Young et al., 2019). It is possible that toxic compounds in foxglove nectar are differentially toxic to particular visiting species, for example generalist robbers, influencing whether they can feed on the plant as has been seen in other species (Barlow et al., 2017; Villalona et al., 2020). Further work into the potential role of nectar toxicity and other floral traits and how their relationship with fitness changes under different intensities of nectar robbing in *D*. *purpurea* could give insight into how nectar robbing can affect the trajectory of a plant's evolution.

Our findings contribute to the growing body of evidence that a changed pollination environment, including nectar robbing, can have strong effects on visitation to a plant and the subsequent reproductive output. The addition of novel floral visitors to a plant's assemblage is likely to become more frequent as plants and nectivorous animal ranges shift due to human influence (Cheptou et al., 2017; Valiente‐Banuet & Verdú, 2013). Therefore, it is important to understand how plants are likely to respond or change as a result of addition of new interacting partners. Further studies on this system could examine whether the addition of nectar robbers affects reproductive output in natural populations and how different nectar robbing communities in different parts of the range of *D*. *purpurea* are affecting the plant's evolution.

## CONFLICT OF INTEREST

The authors declare no conflict of interest.

## AUTHOR CONTRIBUTIONS

**Christopher R. Mackin**: Conceptualization (equal); Formal analysis (equal); Investigation (equal); Writing‐original draft (lead); Data curation (equal); Writing‐review & editing (equal); Methodology (equal). **Dave Goulson**: Methodology (equal); Resources (equal); Writing‐review & editing (equal). **Maria Clara Castellanos**: Conceptualization (equal); Formal analysis (equal); Investigation (equal); Writing‐original draft (equal); Data curation (equal); Writing‐review & editing (equal); Supervision (lead); Project administration (equal); Methodology (equal); Resources (equal).

## Data Availability

All data associated with this study have been made available in the Dryad Digital Repository (https://doi.org/10.5061/dryad.4j0zpc8c1).
